# Left Ventricular Systolic Dysfunction in Patients Diagnosed With Hypertrophic Cardiomyopathy During Childhood: Insights From the SHaRe Registry

**DOI:** 10.1161/CIRCULATIONAHA.122.062517

**Published:** 2023-05-25

**Authors:** Sarah Abou Alaiwi, Thomas M. Roston, Peter Marstrand, Brian Lee Claggett, Victoria N. Parikh, Adam S. Helms, Jodie Ingles, Rachel Lampert, Neal K. Lakdawala, Michelle Michels, Anjali T. Owens, Joseph W. Rossano, Sara Saberi, Dominic J. Abrams, Euan A. Ashley, Christopher Semsarian, John C. Stendahl, James S. Ware, Erin Miller, Thomas D. Ryan, Mark W. Russell, Sharlene M. Day, Iacopo Olivotto, Christoffer R. Vissing, Carolyn Y. Ho

**Affiliations:** 1 Department of Medicine, Brigham and Women’s Hospital, Boston, MA (S.A.A., T.M.R., B.L.C., N.K.L., C.Y.H.).; 2 University of British Columbia, Vancouver, Canada (T.M.R.).; 3 Department of Cardiology, Herlev-Gentofte Hospital, Copenhagen University Hospital, Denmark (P.M.).; 4 Center for Inherited Cardiovascular Disease, Division of Cardiovascular Medicine, Stanford University School of Medicine, CA (V.N.P., E.A.A.).; 5 Department of Internal Medicine, Division of Cardiovascular Medicine, University of Michigan, Ann Arbor (A.S.H., S.S., M.W.R.).; 6 Centre for Population Genomics, Garvan Institute of Medical Research and University of New South Wales, Sydney, Australia (J.I.).; 7 Center for Cardiovascular Genetics, Department of Cardiology, Boston Children’s Hospital & Harvard Medical School, MA (D.J.A.).; 8 Agnes Ginges Centre for Molecular Cardiology at Centenary Institute, University of Sydney, Australia (C.S.).; 9 Department of Medicine, Section of Cardiovascular Medicine, Yale University School of Medicine, New Haven, CT (R.L., J.C.S.).; 10 Department of Cardiology, Thoraxcenter, Erasmus Medical Center Rotterdam, the Netherlands (M.M.).; 11 Division of Cardiology, University of Pennsylvania, Philadelphia (A.T.O., S.M.D.).; 12 Division of Cardiology, Children’s Hospital of Philadelphia, Perelman School of Medicine at the University of Pennsylvania, Philadelphia (J.W.R.).; 13 Royal Brompton & Harefield Hospitals, Guy’s and St Thomas’ NHS Foundation Trust, London, UK (J.S.W.).; 14 Department of Pediatrics, University of Cincinnati College of Medicine, OH (E.M., T.D.R.).; 15 Division of Cardiology, The Heart Institute, Cincinnati Children’s Hospital Medical Center, OH (E.M., T.D.R.).; 16 Meyer Children Hospital, Department of Experimental and Clinical Medicine, University of Florence, Italy (I.O.).; 17 Department of Cardiology, Rigshospitalet, Copenhagen University Hospital, Denmark (C.R.V.).

**Keywords:** cardiomyopathies, cardiomyopathy, hypertrophic, genetics, heart failure, ventricular dysfunction

## Abstract

The development of left ventricular systolic dysfunction (LVSD) in hypertrophic cardiomyopathy (HCM) is rare but serious and associated with poor outcomes in adults. Little is known about the prevalence, predictors, and prognosis of LVSD in patients diagnosed with HCM as children.

**METHODS:**

Data from patients with HCM in the international, multicenter SHaRe (Sarcomeric Human Cardiomyopathy Registry) were analyzed. LVSD was defined as left ventricular ejection fraction <50% on echocardiographic reports. Prognosis was assessed by a composite of death, cardiac transplantation, and left ventricular assist device implantation. Predictors of developing incident LVSD and subsequent prognosis with LVSD were assessed using Cox proportional hazards models.

**RESULTS:**

We studied 1010 patients diagnosed with HCM during childhood (<18 years of age) and compared them with 6741 patients with HCM diagnosed as adults. In the pediatric HCM cohort, median age at HCM diagnosis was 12.7 years (interquartile range, 8.0–15.3), and 393 (36%) patients were female. At initial SHaRe site evaluation, 56 (5.5%) patients with childhood-diagnosed HCM had prevalent LVSD, and 92 (9.1%) developed incident LVSD during a median follow-up of 5.5 years. Overall LVSD prevalence was 14.7% compared with 8.7% in patients with adult-diagnosed HCM. Median age at incident LVSD was 32.6 years (interquartile range, 21.3–41.6) for the pediatric cohort and 57.2 years (interquartile range, 47.3–66.5) for the adult cohort. Predictors of developing incident LVSD in childhood-diagnosed HCM included age <12 years at HCM diagnosis (hazard ratio [HR], 1.72 [CI, 1.13–2.62), male sex (HR, 3.1 [CI, 1.88–5.2), carrying a pathogenic sarcomere variant (HR, 2.19 [CI, 1.08–4.4]), previous septal reduction therapy (HR, 2.34 [CI, 1.42–3.9]), and lower initial left ventricular ejection fraction (HR, 1.53 [CI, 1.38–1.69] per 5% decrease). Forty percent of patients with LVSD and HCM diagnosed during childhood met the composite outcome, with higher rates in female participants (HR, 2.60 [CI, 1.41–4.78]) and patients with a left ventricular ejection fraction <35% (HR, 3.76 [2.16–6.52]).

**CONCLUSIONS:**

Patients with childhood-diagnosed HCM have a significantly higher lifetime risk of developing LVSD, and LVSD emerges earlier than for patients with adult-diagnosed HCM. Regardless of age at diagnosis with HCM or LVSD, the prognosis with LVSD is poor, warranting careful surveillance for LVSD, especially as children with HCM transition to adult care.

**Clinical Perspective**

**What Is New?**

- Patients with hypertrophic cardiomyopathy (HCM) diagnosed during childhood are at greater lifetime risk of developing left ventricular systolic dysfunction (LVSD) compared with patients diagnosed with HCM as adults.
- Despite diagnosis with HCM during childhood, LVSD typically develops during adulthood and is associated with adverse outcomes.
- Risk factors for developing LVSD include younger age at diagnosis of HCM, male sex, low normal initial left ventricular ejection fraction, and carrying pathogenic variants in genes encoding sarcomere proteins.
- Female sex and ejection fraction <35% portend worse prognostic outcomes for patients with childhood presentation of HCM who develop LVSD.

**What Are the Clinical Implications?**

- Clinicians should be aware of the higher risk for developing LVSD in patients diagnosed with HCM in childhood and follow patients accordingly.
- Heightened awareness is particularly relevant because LVSD is likely to manifest during adulthood, when most of these patients have transitioned care from their original pediatric providers to adult cardiologists.

Hypertrophic cardiomyopathy (HCM) is a primary myocardial disorder resulting in left ventricular hypertrophy (LVH) in the absence of an alternative pathology.^1^ The prevalence of adult HCM is estimated at 1 per 500,^1^ but HCM is far less frequent in pediatric populations, with an estimated prevalence of 0.5 to 3 per 100 000.^2–4^ Therefore, our understanding of HCM almost exclusively reflects the experience of adults. Recent studies from SHaRe (Sarcomeric Human Cardiomyopathy Registry) and from the United Kingdom indicate that the natural history of pediatric HCM differs from that of adult HCM.^5,6^ Children diagnosed with HCM have a higher burden of ventricular arrhythmias earlier in life, and often develop atrial fibrillation (AF) and heart failure (HF) in the fourth or fifth decades of life.^5^ Pathogenic variants in genes encoding sarcomere proteins are significantly more likely to be found in a child with HCM than in an adult, and confer a >2-fold increased risk of developing HF.^5^ In contrast, HCM diagnosed during adulthood is more likely to be caused by polygenic or nongenetic disease and is dominated by later-onset AF and HF rather than ventricular arrhythmias.^7,8^ Moreover, conventionally accepted risk factors for poor outcomes in adults with HCM may not be directly applicable to pediatric HCM, as is seen with risk of sudden cardiac death.^9,10^ However, previous studies have not addressed left ventricular systolic dysfunction (LVSD), an adverse consequence of HCM, in children. LVSD, defined as left ventricular ejection fraction (LVEF) <50%, develops in ≈8% of adults with HCM and carries a poor prognosis, with high incidence of death, cardiac transplantation, or left ventricular assist device (LVAD) implantation.^11^ Despite children having the longest lifetime exposure to the abnormal myocardial pathophysiology associated with HCM, very little is known about the prevalence, predictors, and prognosis of LVSD in patients diagnosed with HCM during childhood. In this study, we sought to address these key questions by leveraging multicenter data in SHaRe.

## METHODS

### Study Design

A multicenter observational study with retrospective and prospective elements was performed in SHaRe. The SHaRe consortium maintains a longitudinal database of patients with HCM followed at 13 international, high-volume, expert HCM centers. Aggregated data include historical events before SHaRe entry, demographic data, genetic profiles, clinical phenotypes, and longitudinal, prospective assessment of outcomes, as previously described.^8^ Institutional review board and ethics approval was obtained in accordance with local policies at each SHaRe site. Because of the sensitive nature of the data collected for this study, the data that support the findings of this study are available from the corresponding author upon reasonable request. The statistical code can be accessed through the github repository at https://github.com/christoffervi/childhood_hcm_lvsd.

### Population

The diagnosis of HCM was site-designated and based on standard criteria,^1^ including a maximal left ventricle wall thickness of ≥13 mm (or body surface area–adjusted *Z* score >2 for children), taking into consideration noncardiac findings, genotype, and family history, and allowing experienced clinicians to make an informed diagnosis. Patients were included if they had at least 1 clinic visit at a SHaRe site since 1960 and >1 echocardiographic assessment of left ventricle wall thickness. Childhood-diagnosed HCM was defined as HCM diagnosis before 18 years of age. Clinically reported echocardiographic measurements performed at experienced specialty HCM centers were used for all analyzed metrics of cardiac dimensions and function. Genetic testing was performed at sites using different platforms over time based on availability and clinical practice patterns. Patients with at least one pathogenic or likely pathogenic (P/LP) variant in 1 of 8 major genes encoding components of the sarcomere (*MYBPC3*, *MYH7*, *TNNT2*, *TNNI3*, *TPM1*, *MYL2*, *MYL3*, and *ACTC*)^12^ were designated as having sarcomeric HCM; patients carrying variants of uncertain significance in 1 of the 8 sarcomere genes (as defined by ACMG criteria) were designated as variants of uncertain significance carriers; and patients with benign or likely benign variants or no variants in these genes were defined to have nonsarcomeric HCM. If genetic testing was not performed, patients were classified as being of unknown genetic status. All reported sarcomere variants were classified based on the criteria devised by the American College of Medical Genetics and Genomics and Association for Molecular Pathology.^13^ A subgroup of 3 investigators (S.M.D., J.I., and J.S.W.) adjudicated and standardized the classification of variants that remained ambiguous despite these criteria. Patients were excluded if they had potentially pathogenic variants in genes encoding nonsarcomere cardiac proteins (indicating the presence of a metabolic or storage disease), or an HCM phenocopy, including hypertensive heart disease, Danon disease, Fabry disease, or cardiac amyloidosis.

### Clinical Outcomes

LVSD was defined as an LVEF <50%, as documented on clinical echocardiographic reports (for information on the reliability and reproducibility of the clinically reported LVEF, see the Expanded Methods in the Supplemental Material). Patients were followed from their first visit at a SHaRe site until last follow-up or until meeting the composite outcome of all-cause mortality, cardiac transplantation, or LVAD. Patients with prevalent LVSD were defined as having LVSD at initial SHaRe site evaluation. Patients who developed LVSD during follow-up were defined as having incident LVSD. Analyses to identify factors associated with the development of incident LVSD excluded patients with prevalent LVSD. For all patients with LVSD, age at LVSD recognition was estimated as the age at the first documented LVEF <50% at a SHaRe site.

### Statistical Analyses

SHaRe data through June 2022 were analyzed. Continuous variables are presented as mean±SD if normally distributed or as median (interquartile range [IQR]) if deviating substantially from the normal distribution as evaluated by quantile–quantile plots. Categorical variables are presented as counts and percentages. Between-group comparisons were evaluated statistically using Welch *t* test, Wilcoxon rank sum test, Fisher exact test, or χ^2^ tests as appropriate. Time-to-event analysis was performed to evaluate timing of LVSD recognition (including only patients with incident LVSD [ie, excluding patients with prevalent LVSD], and starting follow-up from the first echocardiogram performed at a SHaRe site) and timing of composite outcome of death, cardiac transplantation, or implantation of LVAD (including all patients with LVSD and using age as the time scale with left-truncation at time of initial recognition of LVSD at a SHaRe site). Time-to-event analyses included use of the Kaplan-Meier method and cumulative incidence function to evaluate event-free survival/cumulative incidence of events and (multivariable) Cox proportional hazards modeling was performed to evaluate the association of baseline variables on the rate and timing of the events of interest. Cox regression results are reported using adjusted hazard ratios (HRs) from multivariable analyses. We confirmed that the proportional hazards assumption was met by scaled Schoenfeld residuals with time. When modeling the composite outcome, septal reduction therapy and having an LVEF <35% were evaluated as time-varying covariates. Age-specific incidence rates of the composite outcome in patients with childhood-diagnosed HCM were calculated according to presence of LVSD. Age-specific incidence rates were reported according to 5 age groups (<12, 12–19, 20–29, 30–39, and >40 years of age). When comparing the overall incidence of the composite outcome between patients with and without LVSD, age standardization between the groups was performed. We used the full population of patients enrolled in the SHaRe Registry (HCM diagnosed in childhood or adulthood) as the reference population. The rates of developing LVSD and the composite outcome were compared between patients with HCM diagnosed in childhood versus adulthood. A *P* value of <0.05 was considered significant. Statistical analyses were conducted using R version 4.1.1 (R Foundation for Statistical Computing).

## RESULTS

### Clinical Characteristics and Prevalence of LVSD in Patients with Childhood-Diagnosed HCM

This study focused on 1010 patients diagnosed with HCM in childhood. Demographic characteristics at initial SHaRe evaluation are presented in the Table 1. The median age at HCM diagnosis was 12.7 years (IQR, 8.0–15.3), and the distribution of age at diagnosis is shown in Figure S1. The median age at initial evaluation at a SHaRe site was 16.5 years (IQR, 12.0–24.4). A total of 65% (n=652) of the cohort were male, and 88% were probands (n=883). Median follow-up duration was 4.9 years (IQR, 2.0–9.2). At the initial SHaRe site visit, LVSD was present in 56 patients (5.5%; prevalent LVSD). Mean LVEF was 67% in those without LVSD compared with 38% in those with prevalent LVSD (*P*<0.001). Patients with prevalent LVSD were of similar age at HCM diagnosis but were significantly older at first SHaRe evaluation (median 31 versus 15 years for prevalent LVSD and no LVSD, respectively; *P*<0.001) and were more likely to report severe HF symptoms (New York Heart Association class III or IV in 36% versus 7%, respectively; *P*<0.001; Table 1). Genetic testing was performed on 664 patients (66%) and had a diagnostic yield of 63% (419 patients) for P/LP variants in sarcomere genes. The yield of genetic testing was not significantly different in patients with or without prevalent LVSD.

**Table 1.**
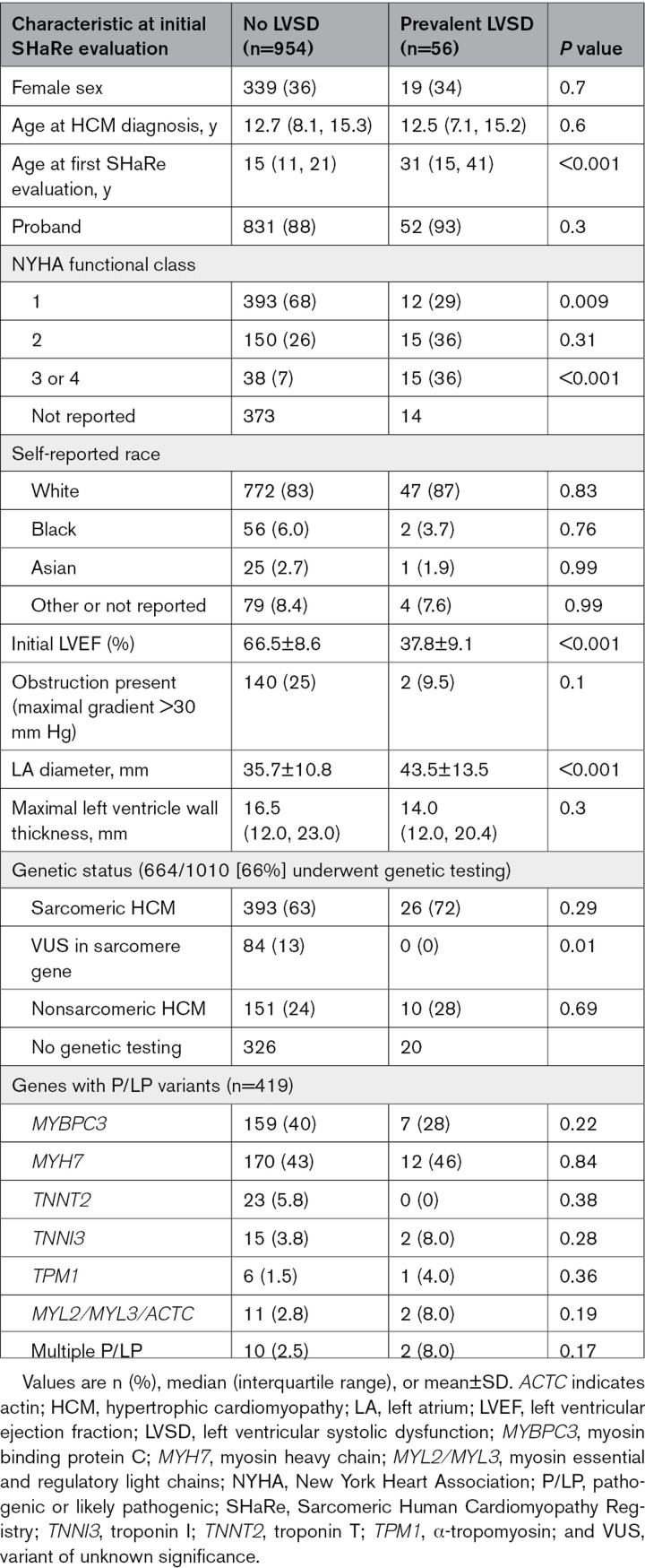
Clinical Characteristics of Patients With Childhood-Diagnosed Hypertrophic Cardiomyopathy, Stratified by Left Ventricular Systolic Dysfunction Status

### Incidence and Predictors of Developing LVSD in Patients with Childhood-Diagnosed HCM

**Figure 1.**
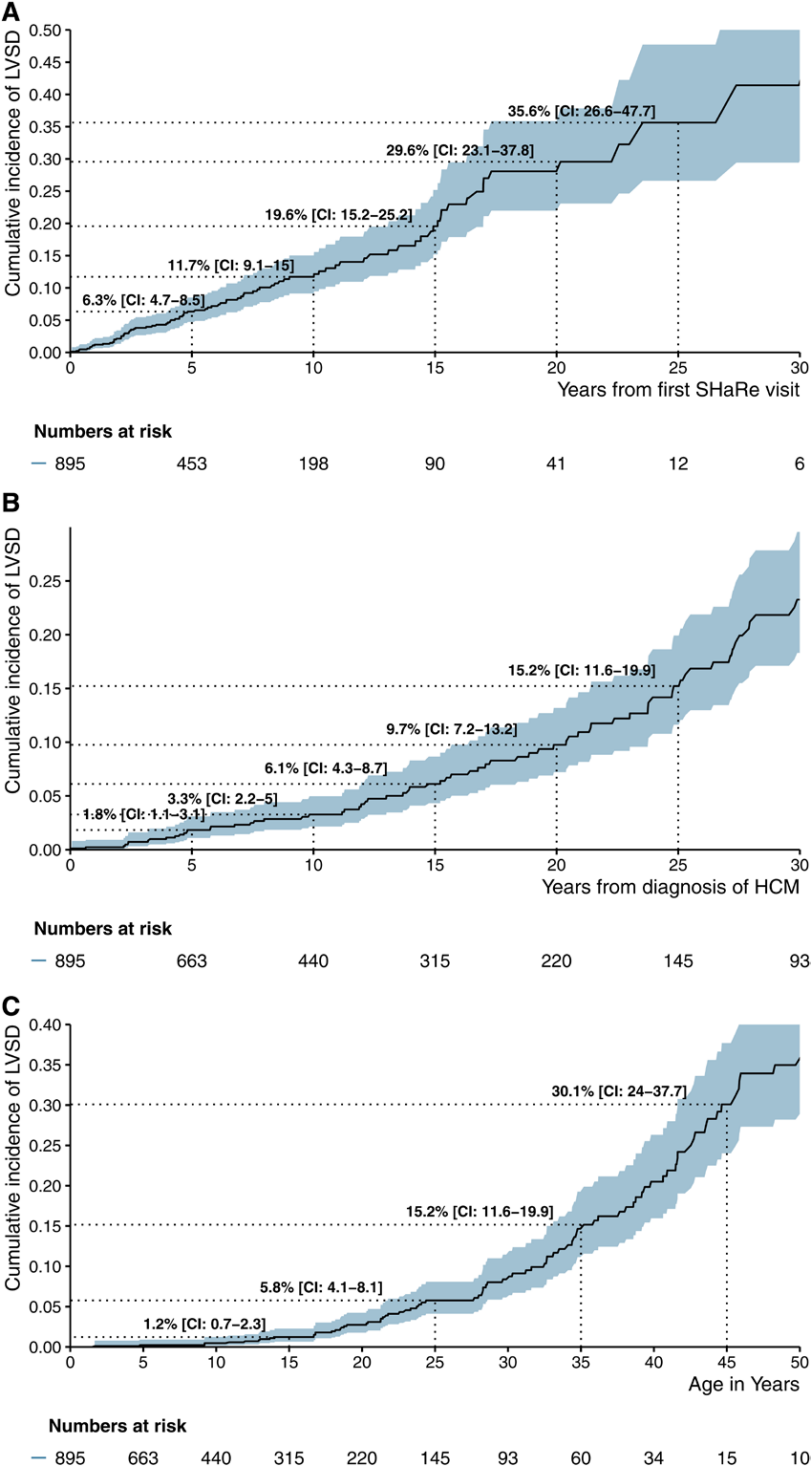
**Cumulative incidence of left ventricular systolic dysfunction in patients with childhood-diagnosed hypertrophic cardiomyopathy.** Cumulative incidence of left ventricular systolic dysfunction (LVSD) in patients with childhood-diagnosed hypertrophic cardiomyopathy (HCM) from time (in years) since initial SHaRe (Sarcomeric Human Cardiomyopathy Registry) evaluation (**A**), time (in years) since diagnosis with HCM (**B**), and birth (age in years; **C**). Of the 954 patients with childhood-diagnosed HCM and no left ventricular systolic dysfunction at initial SHaRe visit, 59 patients did not have follow-up.

During 5920 person-years of follow-up (median, 5.5 years) on 895 patients with longitudinal evaluation and without prevalent LVSD, 92 (10.3%) developed LVSD (incident LVSD). This corresponds to an overall rate of developing LVSD of 16 (CI, 13–19) per 1000 person-years for patients with childhood-diagnosed HCM. The estimated cumulative incidence of LVSD was 11.7% (CI, 9.2–15.1) at 10 years of follow-up (Figure 1A), and LVSD incidence increased with age (Figure S2). Figure 1B and 1C provide cumulative incidence of LVSD as a function of time from HCM diagnosis and age, respectively.

In total, 148 patients with childhood-diagnosed HCM had prevalent or incident LVSD, corresponding to an overall prevalence of LVSD of 14.7% in this cohort. In most patients (82% [122/148]), LVSD was identified after 18 years of age, with a median age at LVSD recognition of 32.7 years (IQR, 21.3–42.1).

**Figure 2.**
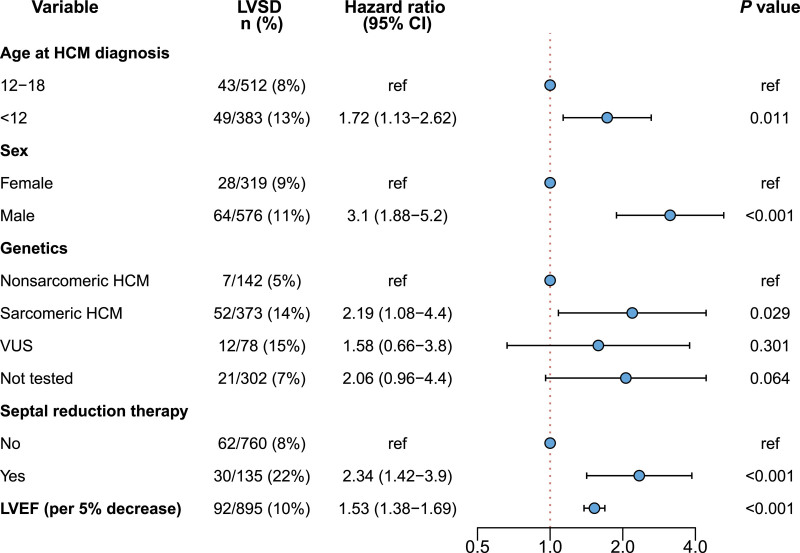
**Predictors of developing incident left ventricular systolic dysfunction in patients with childhood-diagnosed hypertrophic cardiomyopathy, from the time of first SHaRe Registry evaluation.** Patients with prevalent left ventricular systolic dysfunction (LVSD) or with missing values on left ventricular ejection fraction (LVEF) at initial evaluation were excluded. Results are reported from a multiple Cox proportional hazards model. HCM indicates hypertrophic cardiomyopathy; P/LP, pathogenic or likely pathogenic; SHaRe, Sarcomeric Human Cardiomyopathy Registry; and VUS, variant of unknown significance.

Clinical variables were analyzed to identify predictors of developing incident LVSD (Figure 2). Patients with prevalent LVSD were excluded from this analysis. HCM diagnosis before 12 years of age (HR, 1.72 [CI, 1.13–2.62]), male sex (HR, 3.1 [CI, 1.88–5.2]), sarcomeric HCM (HR, 2.19 [CI, 1.08–4.4]), previous septal reduction therapy (HR, 2.34 [CI, 1.42–3.9]), and lower LVEF at initial SHaRe visit (HR, 1.53 [CI, 1.38–1.69] per 5% decrease) were significantly associated with an increased hazard of developing LVSD during follow-up. Further stratifying genetic subtypes of sarcomeric HCM, the risk was highest in patients who carried multiple P/LP variants (HR, 8.32 [CI, 2.11–32.88]) or variants in thick filament genes (HR, 2.25 [CI, 1.1–4.63]) compared with patients with nonsarcomeric HCM (Figure S3). Given the association between younger age at HCM diagnosis and LVSD, we explored the clinical trajectory of patients with a very early presentation of HCM and the age-specific incidence rates of LVSD in childhood (Figure S4A and S4B). Overall, we found similar cumulative incidence rates of LVSD in patients diagnosed with HCM before 2 years of age compared with other pediatric groups, and that the age-specific incidence of LVSD is low throughout childhood. However, the power to detect differences in these subgroups is limited.

### Clinical Outcomes in Patients With LVSD and Childhood-Diagnosed HCM

**Figure 3.**
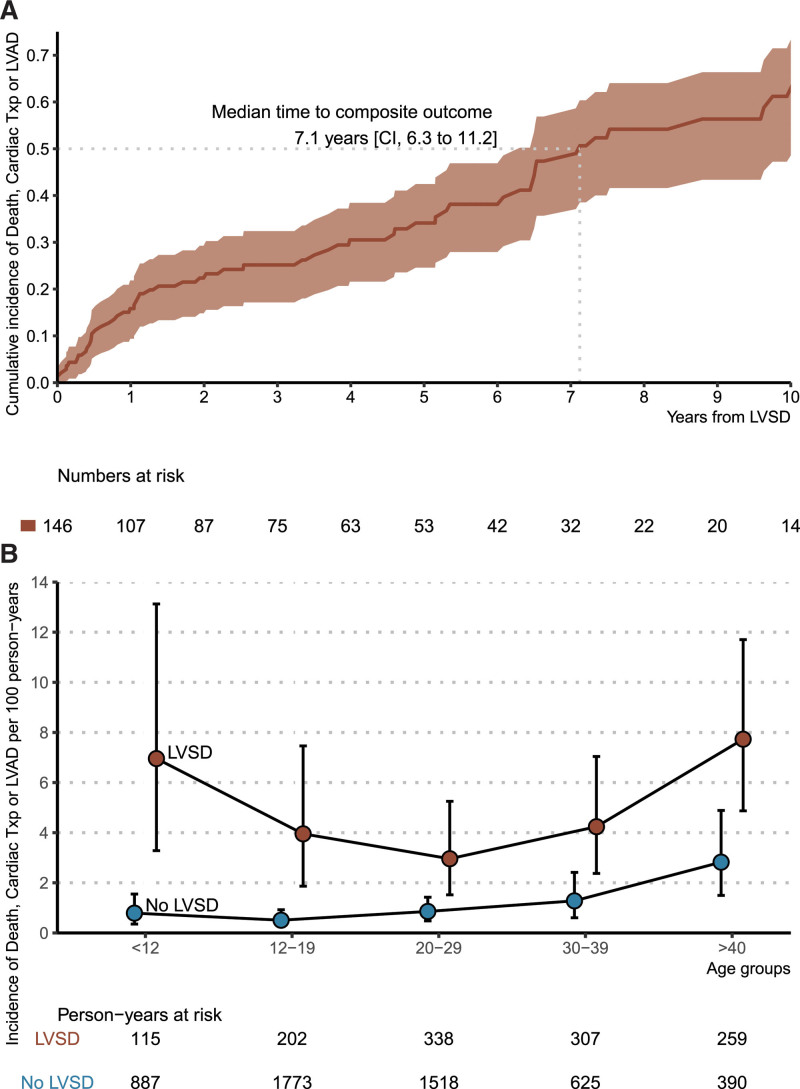
**Cumulative and age-specific incidence of major adverse outcomes in patients with childhood-diagnosed hypertrophic cardiomyopathy stratified by the presence of left ventricular systolic dysfunction. A**, Cumulative incidence of death, cardiac transplantation (**Txp**), or left ventricular assist device (LVAD) implantation in patients with childhood diagnosis of hypertrophic cardiomyopathy and left ventricular systolic dysfunction (LVSD). **B**, Age-specific incidence of death, **Txp**, or LVAD implantation in patients with childhood-diagnosed hypertrophic cardiomyopathy stratified by the presence of LVSD. The error bars denote the 95% CIs, and the groups have been slightly offset in the horizontal plane to avoid overlap.

We next focused on all patients with childhood-diagnosed HCM and LVSD (n=148) to characterize the prognosis of LVSD and to identify predictors of adverse outcomes. Table S1 lists major outcomes in patients with and without LVSD. Of 148 patients with LVSD, 59 (40%) developed the composite end point of cardiac transplantation (n=38), LVAD implantation (n=5), or death (n=24; 8 from HF, 9 from sudden cardiac death). The median age at transplant was 37.9 years (IQR, 19.7–47.7), and the median age at death was 37 years (IQR, 21.7–52.1). The median time from LVSD recognition to the composite end point was 7.1 years (CI, 6.3–11.2; Figure 3A). For a descriptive characterization of the timing of LVSD identification and the development of the composite outcomes relative to the time of HCM diagnosis in patients with childhood-diagnosed HCM, see Figure S5. Compared with individuals with normal LVEF, LVSD portended worse prognosis across all age groups, as indicated by overall age-standardized composite outcome incidence rates of 4.6 (CI, 3.3–6.2) versus 1.0 (CI, 0.7–1.3) per 100 person-years. This difference was especially pronounced in the oldest and youngest age groups (Figure 3B).

**Figure 4.**
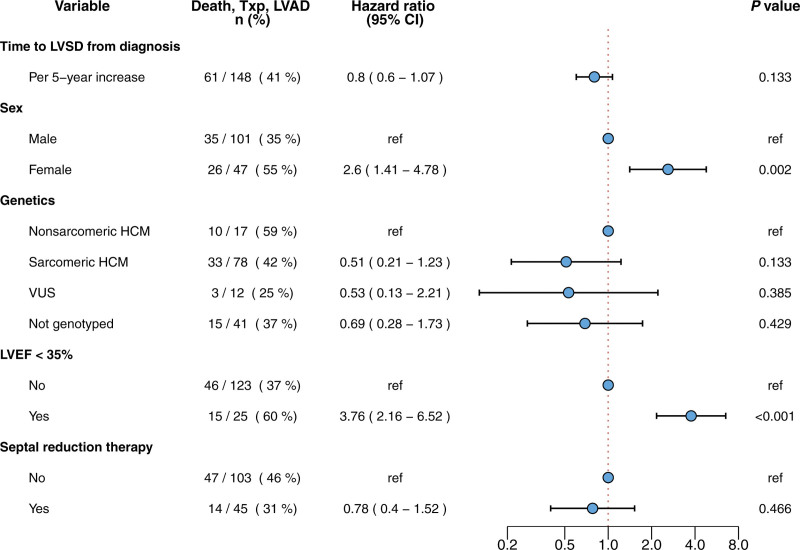
**Predictors of the primary outcome of death, cardiac transplantation, or left ventricular assist device implantation in patients with childhood-diagnosed hypertrophic cardiomyopathy who develop left ventricular systolic dysfunction.** Results are reported from a multiple Cox proportional hazards model, in which age was used as the time scale with left-truncation at time of left ventricular systolic dysfunction. LVAD indicates left ventricular assist device; LVEF, left ventricular ejection fraction; LVSD, left ventricular systolic dysfunction; Txp, cardiac transplantation; and VUS, variant of unknown significance.

Female sex (HR, 2.60 [CI, 1.41–4.78]) and LVEF <35% (HR, 3.76 [CI, 2.16–6.52]) were significantly associated with the primary composite outcome after development of LVSD (Figure 4). The time from HCM diagnosis to LVSD recognition, undergoing septal reduction therapy, and carrying sarcomere variants were not significantly associated with the primary outcome (Figure 4).

To assess the potential confounding effect of septal reduction therapy in modifying the natural history of left ventricular function, sensitivity analyses were performed, censoring patients at the time of septal reduction therapy. These analyses yielded consistent results for predicting both incidence and outcomes of LVSD, suggesting that septal reduction therapy is less likely to be a confounding factor in our analysis (Figures S6 and S7). In addition, given the potential effect of relatedness on our results and the suggested difference between probands and non-proband family members (≈13% of our total cohort), we performed a proband-only sensitivity analysis (Figures S8 and S9). The findings supported the results found in the overall cohort.

### LVSD in Patients With HCM Diagnosed During Childhood Versus Adulthood

**Figure 5.**
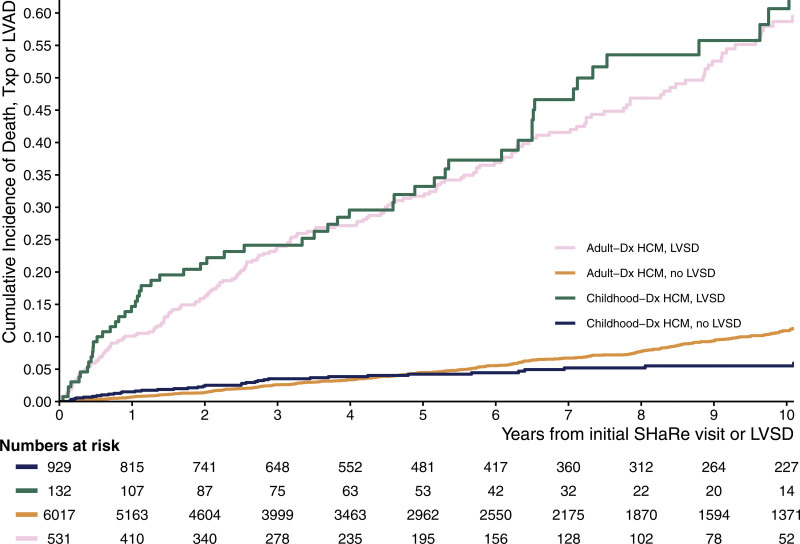
**Cumulative incidence of the composite outcome of death, cardiac transplant, or left ventricular device implantation from time of clinical recognition of left ventricular systolic dysfunction.** The figure includes patients with prevalent and incident left ventricular systolic dysfunction (LVSD). HCM indicates hypertrophic cardiomyopathy; LVAD, left ventricular assist device; SHaRe, Sarcomeric Human Cardiomyopathy Registry; and Txp, cardiac transplantation.

To investigate the effect of the age at HCM diagnosis on the development and outcomes of LVSD, we compared SHaRe participants diagnosed with HCM during childhood with those diagnosed with HCM during adulthood (n=6741; Table S2). The overall prevalence of LVSD was higher in patients with childhood-diagnosed HCM compared with those with adult-diagnosed HCM (14.7% versus 8.8%; odds ratio, 1.78 [95% CI, 1.45–2.16]; *P*<0.0001). The median age of developing LVSD was 58 years (IQR, 47.2–67.0) in adult-diagnosed HCM compared with 32.6 years (IQR, 21.4–41.5) in childhood-diagnosed HCM. Despite the higher prevalence of LVSD in childhood-diagnosed HCM, prognosis was similar, with no significant difference detected in the incidence (40% versus 36%; *P*=0.22) or rate (HR, 1.13 [CI, 0.85–1.5]; *P*=0.41) of the composite outcome of death, cardiac transplantation, or LVAD implantation (Figure 5).

## DISCUSSION

In this observational multicenter study, we described the prevalence, prognosis, and predictors of LVSD in 1010 patients in SHaRe who were diagnosed with HCM as children. The overall prevalence of LVSD was 14.7%, with an overall incidence rate of 1.6% (CI, 1.3–1.9%) per person-year. Despite childhood diagnosis of HCM, LVSD typically was not recognized until adulthood (median age, 32.6 years [21.3–41.6]). Younger age at diagnosis, lower initial LVEF, male sex, pathogenic sarcomere gene variants, and previous invasive septal reduction therapy were independently associated with a higher risk of developing LVSD. The prognosis after developing LVSD was poor; 40% of patients developed advanced HF, underwent cardiac transplantation, received LVAD, or died within a median of 7.1 years after developing LVSD. Predictors of adverse outcomes included LVEF <35% and female sex. In addition, we demonstrated that LVSD was almost twice as prevalent in patients diagnosed with HCM as children compared with those diagnosed as adults, for whom the overall prevalence of LVSD is ≈8%, based on our current work and published literature.^8,14–18^

Important differences have been described between patients diagnosed with HCM during childhood and adulthood.^5^ Patients diagnosed as children are more likely to carry pathogenic sarcomere variants and are at increased risk of ventricular arrhythmias and advanced HF.^5^ LVSD complicating early-diagnosed HCM has not been well-characterized. Previous studies have followed smaller numbers of children (80–687 patients) for shorter periods of time and with less extensive clinical detail.^6,19–21^ Furthermore, there has been a limited understanding about whether age at HCM diagnosis is relevant to the natural history of the disease and contributes to the risk of LVSD development. Our findings, coupled with previous work, collectively indicate that patients with LVSD were more likely to be diagnosed with HCM as children. This study also demonstrates that patients with HCM who develop LVSD, whether diagnosed in adulthood or childhood, share similar clinical and genetic characteristics, including the presence of a sarcomere variant (particularly multiple variants) and lower LVEF at initial evaluation. In addition, although patients diagnosed with HCM in childhood were more likely to develop LVSD, the prognosis related to LVSD appeared similar, irrespective of age at HCM diagnosis, both in SHaRe and previous smaller studies (median survival 2.4–8.4 years).^11,14–18^

This study highlights several characteristics pertinent to patients who are diagnosed with HCM during childhood and who develop LVSD later in life. First, male patients were twice as likely to develop LVSD but were half as likely to die or require LVAD support or cardiac transplantation after clinical recognition of LVSD. This association was not apparent in the adult cohort.^11^ In addition, there were several genetic findings of interest. Children with multiple P/LP sarcomere variants had more than an 8-fold greater risk for developing incident LVSD. This is consistent with previous data showing that patients with multiple sarcomere variants are more severely affected by HCM.^8,22,23^ In this cohort of patients with childhood-diagnosed HCM, variants in genes encoding thick filament proteins (*MYH7*, *MYBPC3*, *MYL2*, and *MYL3*) were associated with a 2-fold increase in the risk of incident LVSD. This adds to the existing literature correlating P/LP variants in thick filaments (particularly in *MYH7* and *MYBPC3*) with worse outcomes in pediatric HCM.^10,23,24^ However, this finding contrasts with our previous results in adult-diagnosed HCM, in which variants in thin filament genes were associated with a higher risk of incident LVSD.^11^ In a retrospective study of 230 adults with HCM, thin filament–related HCM was associated with a lower prevalence of outflow tract obstruction and a lower maximal wall thickness but a higher predilection for advanced HF, systolic dysfunction, and restrictive physiology compared with thick filament–related HCM.^25^ Although the smaller size of the childhood-diagnosed HCM cohort may have limited our ability to find an association with rarer thin filament variants, our findings collectively provide further support that underlying genotype may have different implications for patients diagnosed with HCM during childhood versus adulthood.

As seen in SHaRe participants with HCM diagnosed during adulthood, previous invasive septal reduction therapy was significantly associated with incident LVSD in childhood-diagnosed HCM. Alcohol septal ablation and surgical myectomy both independently predicted the development of LVSD across all ages.^11^ A correlation between septal reduction therapy and adverse remodeling as well as lower long-term survival compared with the general population has been described in previous small studies.^26,27^ Because myectomy is highly effective for reducing obstructive symptoms in patients with HCM,^28,29^ especially among young individuals, it is important to know whether postprocedural long-term adaptive or maladaptive remodeling may occur. Both in this study and in the previous study on LVSD in adult HCM, septal reduction therapy did not significantly alter the prognosis associated with LVSD.^11^ Although these studies are observational, given these findings, careful longitudinal clinical follow-up after invasive septal reduction is prudent to monitor for incipient LVSD. Whether this association is attributable to the need for septal reduction therapy serving as a marker for more severe disease (ie, intervention performed in patients with more substantial remodeling, and therefore at higher risk for adverse outcomes) or whether there may be a more direct relationship between these procedures and long-term systolic function is unclear. Continued longitudinal surveillance in large cohorts with HCM is needed to understand the association between septal reduction therapy and LVSD.

It is important to recognize that although patients with a childhood diagnosis of HCM have a higher lifetime prevalence of LVSD, findings are most likely to manifest during adulthood, at a stage when most of these patients have transitioned their care to adult cardiology. It is unclear whether the higher prevalence of LVSD in childhood-diagnosed HCM is attributable to disease-specific attributes or longer exposure to disease, or whether it simply reflects a longer duration of follow-up. However, these findings are consistent with previous work demonstrating that the risk of adverse outcomes in childhood-diagnosed HCM extends well beyond adolescence.^21^ Patients with LVSD have worse outcomes than other patients with HCM, so it is crucial for clinicians caring for patients diagnosed with HCM during childhood to be cognizant of the risk. Greater vigilance in screening for LVSD may be appropriate in order to best mobilize clinical intervention, ideally before progression to end-stage disease. The relatively long timeframe from HCM diagnosis to recognition of LVSD also suggests a valuable window of opportunity to implement disease-modifying therapies in high-risk patients.

### Limitations

Limitations of SHaRe are inherent to its pragmatic, real-world, multicenter, partially retrospective observational design, whereby availability of certain assessments, such as cardiac magnetic resonance imaging or genetic testing, varies due to local resources and clinical practice, both of which also change over time. Clinical characteristics at initial HCM diagnosis are sometimes unavailable and only obtained at initial SHaRe site evaluation; as such, the exact timing of certain outcomes that are prevalent at baseline evaluation (eg, LVSD) at a SHaRe site may be unknown. The presence of LVSD was based on clinically reported echocardiographic measurements at expert centers; these are subject to operator bias and were not standardized to a universal protocol or centrally read. Referral bias may be present, as SHaRe sites are high-volume tertiary centers. As with any registry, survival bias is inherent, as patients must have survived to their first clinic visit. The longer duration of follow-up for patients with incident and prevalent LVSD may result in an overestimation of LVSD risk compared with HCM with a normal LVEF. Given the retrospective nature of some data, patients with LVSD may be at risk of ascertainment bias and having more frequent echocardiograms for surveillance of ejection fraction, as they are likely to be considered higher-risk patients by most clinicians. Detailed data on the use and dosing of guideline-directed medical therapy for HF with reduced LVEF are not available in SHaRe, which may alter some prognostic findings. The smaller cohort size in pediatric HCM may limit power and ability to detect significant associations seen in larger cohorts; however, this is one of the largest efforts to date aimed at longitudinally following patients with childhood-diagnosed HCM. Our cohort primarily consists of individuals with White ancestry, and representation of other races and ethnicities is limited. Furthermore, most patients were identified as probands. It is uncertain whether the prognosis of patients diagnosed through family screening might be more favorable. In addition, we intentionally excluded patients with inborn errors of metabolism, glycogen storage disease, and other syndromic causes of left ventricular hypertrophy to focus on the most common form of HCM: sarcomeric HCM. Most studies of pediatric HCM have included children with these and other HCM genocopies.

### Conclusions

Patients diagnosed with HCM during childhood have a higher lifetime risk of developing LVSD and related adverse outcomes than patients diagnosed with HCM as adults. Both LVSD and adverse outcomes are most likely to manifest during early adulthood rather than childhood. Male sex, low normal LVEF, and the presence of sarcomere variants, particularly variants in the thick filament genes *MYH7* and *MYBPC3* and multiple sarcomere gene variants, are independently associated with developing incident LVSD in patients with childhood-diagnosed HCM. Female patients and patients with the lowest presenting LVEF are at highest risk for poor outcomes. Greater recognition of and surveillance for LVSD in patients diagnosed with HCM as children are crucial to provide the best care, especially as these patients transition to adulthood and management by adult cardiologists.

## ARTICLE INFORMATION

### Acknowledgments

The authors thank the site data managers, the patients, and their families.

### Sources of Funding

The SHaRe Registry (Sarcomeric Human Cardiomyopathy) receives unrestricted research support from Bristol Myers Squibb, Pfizer, Biomarin, and Cytokinetics. Dr Ho receives funding from the National Institutes of Health (R01HL155568). Dr Semsarian is the recipient of a National Health and Medical Research Council practitioner fellowship (fellowship 1154992). Dr Ware is supported by the Sir Jules Thorn Charitable Trust (grant 21JTA), UK Medical Research Council, British Heart Foundation (grant RE/18/4/34215), the National Institute for Health and Care Research Imperial College Biomedical Research Centre, and the National Institute for Health and Care Research Royal Brompton Cardiovascular Biomedical Research Unit.

### Disclosures

Dr Ho is a consultant for and receives research funding from Bristol Myers Squib, Pfizer, Cytokinetics, Tenaya, and BioMarin. Dr Parikh receives research funding from BioMarin and consults for Nuevocor and Viz.ai. Dr Lakdawala is a consultant for Bristol Myers Squibb, Pfizer, Cytokinetics, Tenaya, and Sarepta, and receives research funding from Pfizer. Drs Saberi and Michels are consultants for Bristol Myers Squibb and Cytokinetics. Dr Claggett has received personal fees from Amgen, Cardurion, Corvia, Cytokinetics, Intellia, and Novartis outside the submitted work. Dr Ashley is a co-founder of Deepcell, Personalis, and SVEXA, a board member of AstraZeneca, and an adviser to Apple, Foresite Labs, Nuevocor, and Sequencebio. Dr Lampert has received research funding and honoraria from Medtronic and Abbott, honoraria from Boston Scientific, and sat on advisory board for Medtronic. Dr Olivotto is a consultant for or receives research grants from: BMS-Myokardia, Cytokinetics, Boston Scientific, Sanofi Genzyme, Shire Takeda, Amicus, Menarini International, Bayer, and Tenaya. Dr Helms receives research funding from Bristol Myers Squibb and is a consultant for Tenaya and Lexeo Therapeutics. Dr Ingles receives research grant support from Bristol Myers Squibb. Dr Rossano is a consultant for Merck, Bayer, Bristol Myers Squibb, BioMarin, and CRI Biotech. Dr Ware has consulted for MyoKardia (now Bristol Myers Squibb), Foresite Labs, and Pfizer. Dr Abrams is a consultant for Dinaqor. Dr Marstrand has been employed at Novo Nordisk A/S. Novo Nordisk A/S was not involved in this research, and the views presented do not necessarily reflect the views of Novo Nordisk A/S. Drs Alaiwi, Roston, Semsarian, Stendahl, Ryan, Russell, Miller, and Vissing declare no relevant disclosures or competing interests.

### Supplemental Material

Expanded Methods

Tables S1 and S2

Figures S1–S9

## Supplementary Material
