# Supplementary material for: Left Ventricular Systolic Dysfunction in Patients Diagnosed With Hypertrophic Cardiomyopathy During Childhood: Insights From the SHaRe Registry
Source: Circulation. 2023 May 25;148(5):394–404. doi: 10.1161/CIRCULATIONAHA.122.062517 (PMC10373850; doi:10.1161/CIRCULATIONAHA.122.062517)
Supplement: Supplementary file 1 [file cir-148-394-s001.pdf]

SUPPLEMENTAL MATERIAL

Table of contents

Supplemental Methods **2**

Echocardiography *2*

Supplemental Figures: **3**

Supplementary Figure S1: Age at diagnosis of hypertrophic cardiomyopathy *3*

Supplementary Figure S2: Age-specific incidence rates of left ventricular systolic dysfunction *4*

Supplementary Figure S3: Cox proportional hazards model of incident left ventricular systolic dysfunction *5*

Supplementary Figure S4: Incidence of left ventricular systolic dysfunction according to age-groups *6*

Supplementary Figure S5: Chronological timing of HCM diagnosis, occurrence of LVSD and reaching the composite outcome for individual patients *7*

Supplementary Figure S6: Cox model of incident left ventricular systolic dysfunction – sensitivity analysis to investigate the effect of septal reduction therapy *8*

Supplementary Figure S7: Cox model of the composite outcome – sensitivity analysis to investigate the effect of septal reduction therapy *9*

Supplementary Figure S8: Cox model of incident left ventricular systolic dysfunction – sensitivity analysis including only probands *10*

Supplementary Figure S9: Cox model of the composite outcome – sensitivity analysis including only probands *11*

Supplemental Tables: **12**

Supplementary Table S1: Clinical outcomes of patients with childhood-diagnosed hypertrophic cardiomyopathy, stratified by left ventricular systolic dysfunction (LVSD) status. The "All LVSD" group represents patients with both prevalent and incident LVSD. *12*

Supplementary Table S2: Characteristics of patients diagnosed with HCM during childhood or adulthood with and without prevalent LVSD. *13*

Supplemental Methods

Echocardiography

In our cohort, left ventricular ejection fraction (LVEF) was evaluated from clinical echocardiograms performed at each of the 12 high volume HCM referral sites. These studies were performed at hospitals with experienced cardiac imaging clinics and results on LVEF were collected from echocardiography reports, reviewed, and approved by consultant cardiologists specializing in cardiac imaging at each site. The exact method by which LVEF was measured was not standardized and included both the biplane Simpsons method and results from 3D echocardiography, depending on local guidelines and imaging quality. Accordingly, we assessed the reproducibility and reliability of LVEF measurements. In assessing the intra-patient variability in LVEF assessed by echocardiography (with up to 10 examinations per patient), we found a coefficient of variation (CV) of 0.088 corresponding to 5.3%-points. This was similar to the intra-patient CV observed for serial cardiac magnetic resonance (CMR) imaging (0.079 or 5.3%-points). The CV did not vary significantly according to the number of echocardiographic studies performed, or from site to site (range 0.074 to 0.116 or 4.3-6.5 %-points). In patients with an LVEF assessed by both echocardiography and CMR within 3 months of each other, we observed a CV of 0.087 corresponding to 5.6%-points, with a mean difference of 2.2%-points between echocardiography and CMR (lower values for CMR).

Supplemental Figures:

Supplementary Figure S1: **Age at diagnosis of hypertrophic cardiomyopathy**

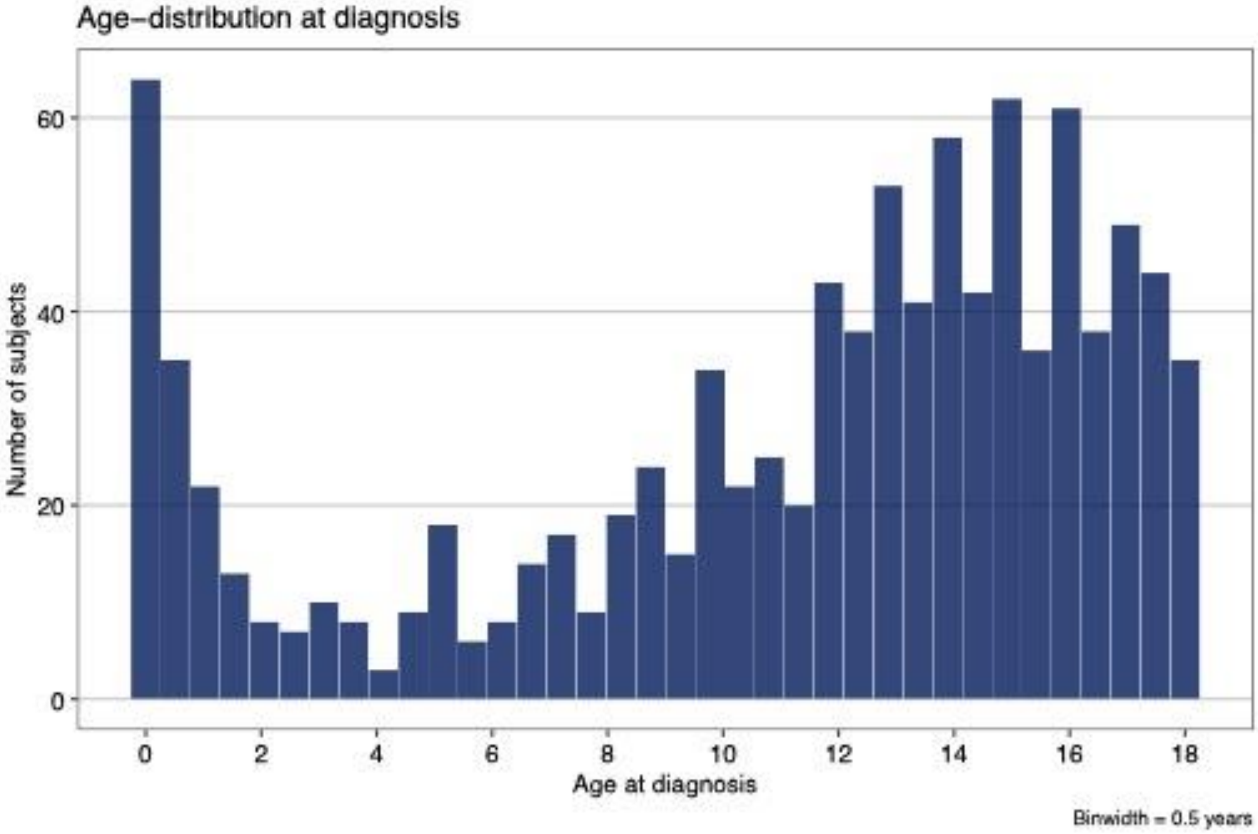

Supplementary Figure S1: Distribution of age at HCM diagnosis in patients with childhood-diagnosed HCM (n=1010)

Supplementary Figure S2: Age-specific incidence rates of left ventricular systolic dysfunction

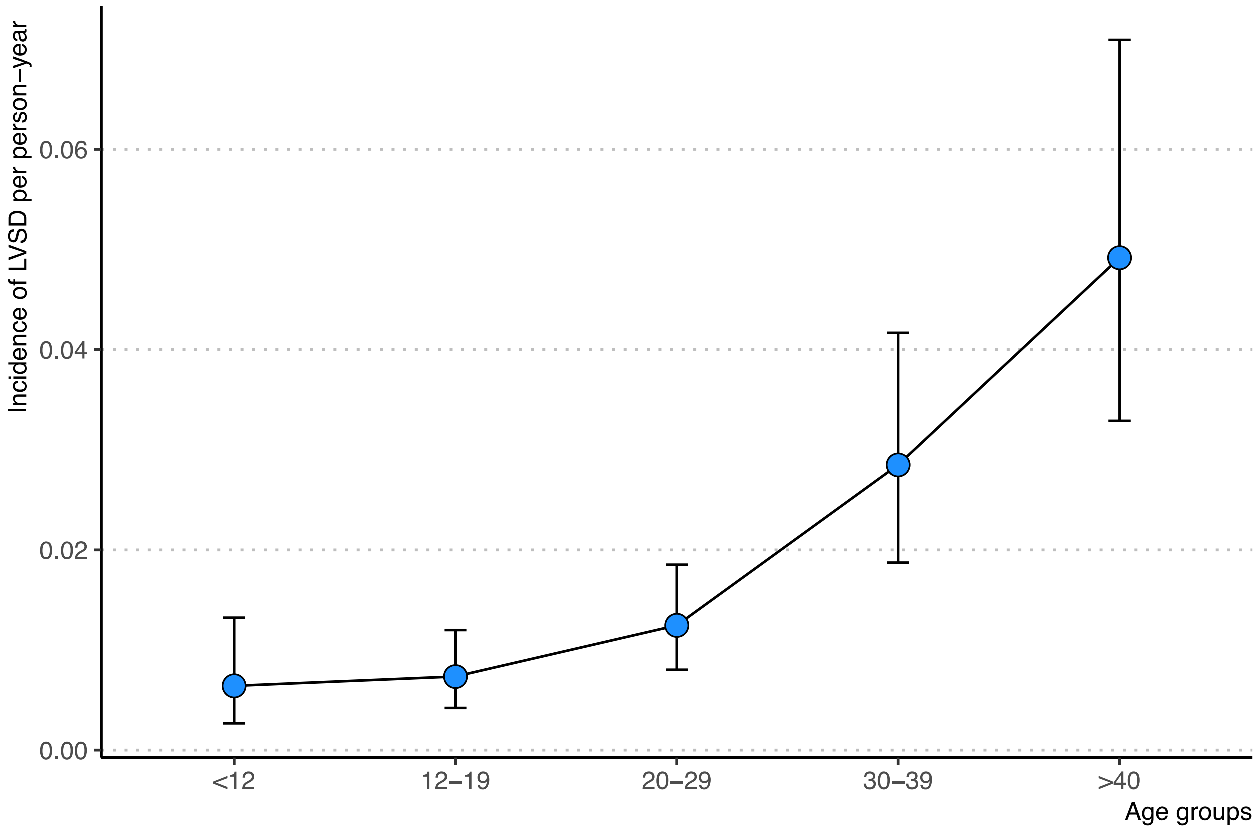

Supplementary Figure S2: Age-specific incidence of developing left ventricular systolic dysfunction in patients diagnosed with HCM during childhood.

Supplementary Figure S3: Cox proportional hazards model of incident left ventricular systolic dysfunction

Variable	LVSD n (%)	Hazard ratio (95% CI)	p–value
**Age at HCM diagnosis**			
12–18	43/512 (8%)	ref	ref
<12	49/383 (13%)	1.74 (1.13–2.67)	0.011
**Sex**			
Female	28/319 (9%)	ref	ref
Male	64/576 (11%)	3.45 (2.04–5.85)	<0.001
**Genetics**			
Non–Sarcomeric HCM	7/142 (5%)	ref	ref
Not tested	21/302 (7%)	2.1 (0.98–4.53)	0.057
VUS	12/78 (15%)	1.6 (0.67–3.83)	0.289
Thick filament P/LP	44/316 (14%)	2.25 (1.1–4.63)	0.027
Thin filament P/LP	5/48 (10%)	1.49 (0.5–4.41)	0.474
Multiple P/LP	3/9 (33%)	8.32 (2.11–32.88)	0.003
**Septal reduction therapy**			
No	62/760 (8%)	ref	ref
Yes	30/135 (22%)	2.37 (1.43–3.93)	<0.001
**LVEF (per 5% decrease)**	92/895 (10%)	1.55 (1.4–1.71)	<0.001

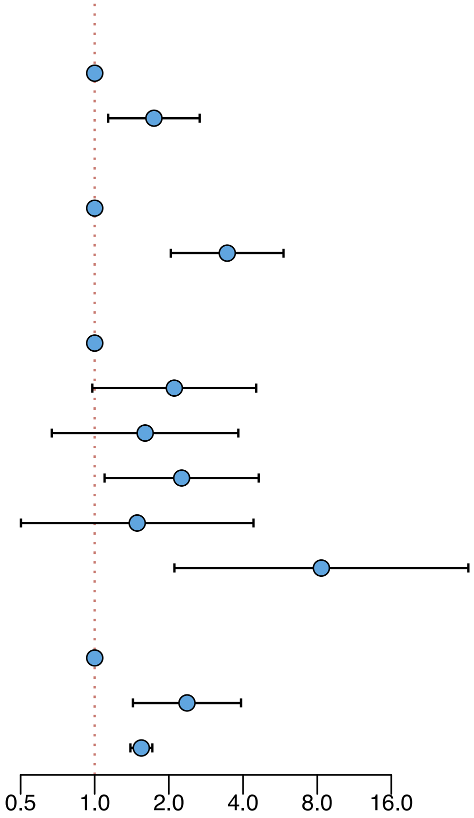

Supplementary Figure S3: Predictors of incident LVSD in children with HCM from the time of first SHaRe visit, further stratifying genetic subtypes. Patients with LVSD at first evaluation or missing values on either wall thickness or LV ejection fraction at initial evaluation were omitted. VUS, variant of unknown significance; P/LP, pathogenic or likely pathogenic.

Supplementary Figure S4: Incidence of left ventricular systolic dysfunction according to age-groups

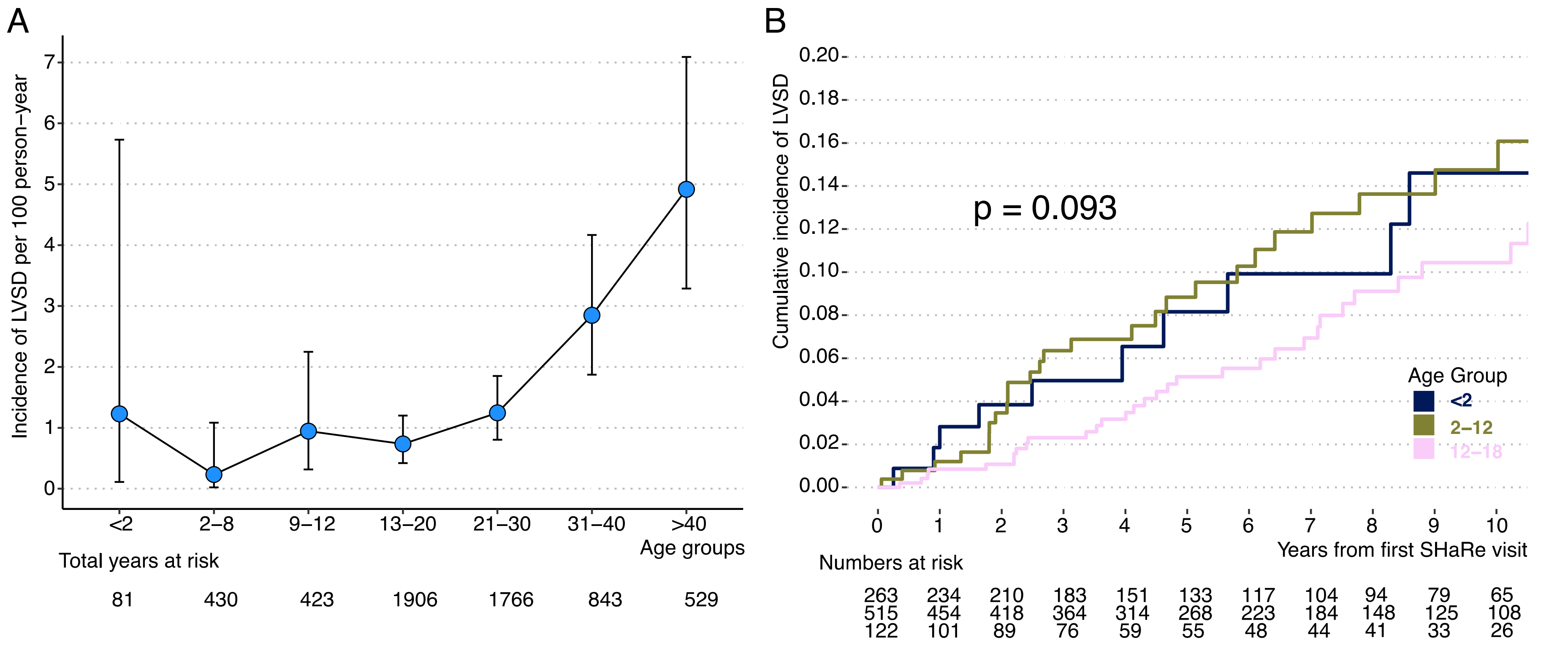

Supplementary Figure S4: Clinical outcomes in patients with very early diagnosis with HCM. A. Age-specific incidence rates of LVSD in patients diagnosed with HCM during childhood. B. The cumulative incidence in patients with HCM diagnosis as infants/toddlers (dark blue), young children (green) and pre-teens/teens (pink).

Supplementary Figure S5: Chronological timing of HCM diagnosis, occurrence of LVSD and reaching the composite outcome for individual patients

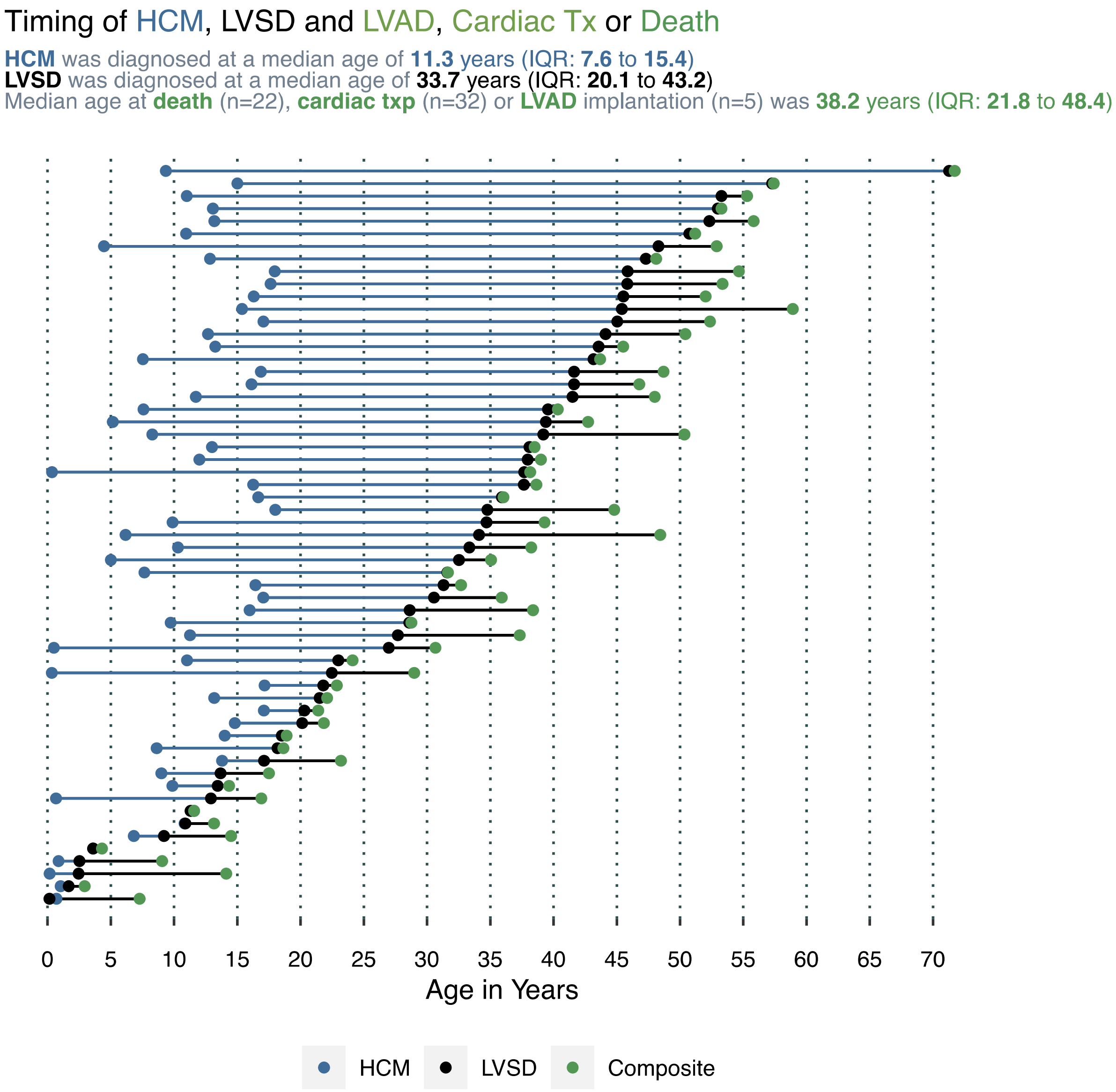

Supplementary Figure S5: Timing of LVSD and composite outcomes relative to the diagnosis of HCM. This figure includes individual patients with childhood-diagnosed HCM, who were recognized to have LVSD and reached the composite outcome in our study. Figure includes patients with prevalent and incident LVSD.

Supplementary Figure S6: Cox model of incident left ventricular systolic dysfunction – sensitivity analysis to investigate the effect of septal reduction therapy

Variable	LVSD n (%)	Hazard ratio (95% CI)	p–value
**Age at HCM**			
12–18	30 / 491 (6 %)	ref	ref
<12	32 / 353 (9 %)	1.99 (1.19 – 3.33)	0.009
**Sex**			
Female	19 / 296 (6 %)	ref	ref
Male	43 / 548 (8 %)	2.27 (1.25 – 4.11)	0.007
**Genetics**			
Non–Sarcomeric HCM	2 / 136 (1 %)	ref	ref
Not Genotyped	15 / 283 (5 %)	4.71 (1.07 – 20.75)	0.04
VUS	7 / 70 (10 %)	4.2 (0.86 – 20.55)	0.076
Sarcomeric HCM	38 / 355 (11 %)	5.43 (1.3 – 22.66)	0.02
**LVEF (per 5% decrease)**	62 / 844 (7 %)	1.62 (1.44 – 1.82)	<0.001

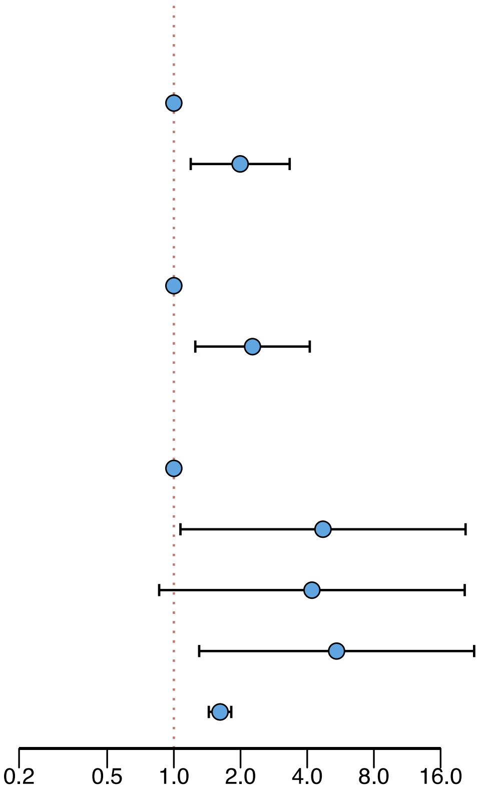

Sensitivity analysis: Subjects with septal reduction therapy censored at time of procedure

Supplementary Figure S6: Predictors of developing incident LVSD in patients with childhood-diagnosed HCM from the time of first SHaRe evaluation with additional sensitivity analysis censoring patients at the time of septal reduction therapy. Patients with LVSD at first evaluation or missing values on either LV wall thickness or ejection fraction at initial evaluation were excluded. VUS, variant of unknown significance.

Supplementary Figure S7: Cox model of the composite outcome – sensitivity analysis to investigate the effect of septal reduction therapy

Variable	Death, Txp, LVAD n (%)	Hazard ratio (95% CI)	p–value
**Time to LVSD from diagnosis**			
Per 5–year increase	61 / 148 (41 %)	0.79 (0.55 – 1.13)	0.191
**Sex**			
Male	35 / 101 (35 %)	ref	ref
Female	26 / 47 (55 %)	2.4 (1.19 – 4.83)	0.015
**Genetics**			
Non–Sarcomeric HCM	10 / 17 (59 %)	ref	ref
Sarcomeric HCM	48 / 119 (40 %)	0.77 (0.3 – 1.97)	0.584
VUS	3 / 12 (25 %)	0.38 (0.04 – 3.55)	0.398
**LVEF < 35%**			
No	46 / 123 (37 %)	ref	ref
Yes	15 / 25 (60 %)	3.52 (1.83 – 6.76)	<0.001

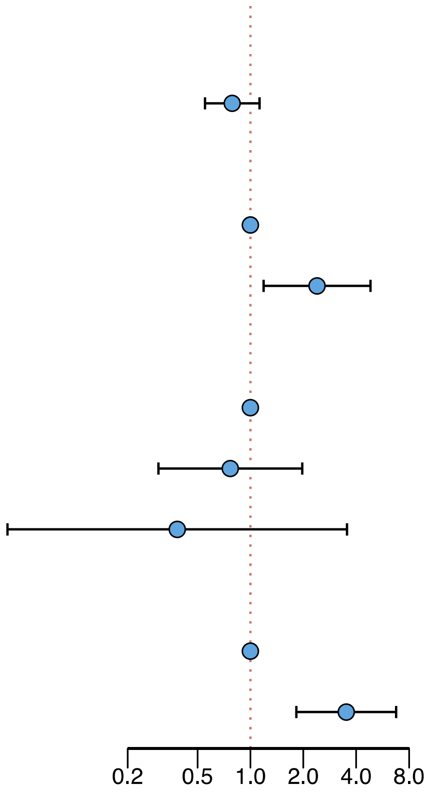

Sensitivity analysis: Subjects with septal reduction therapy censored at time of procedure

Supplementary Figure S7: Predictors of the primary outcome of death, cardiac transplantation (Txp), or left ventricular device (LVAD) implantation in patients diagnosed with HCM during childhood who develop LVSD with additional sensitivity analysis censoring patients at the time of septal reduction therapy. VUS, variant of unknown significance.

Supplementary Figure S8: Cox model of incident left ventricular systolic dysfunction – sensitivity analysis including only probands

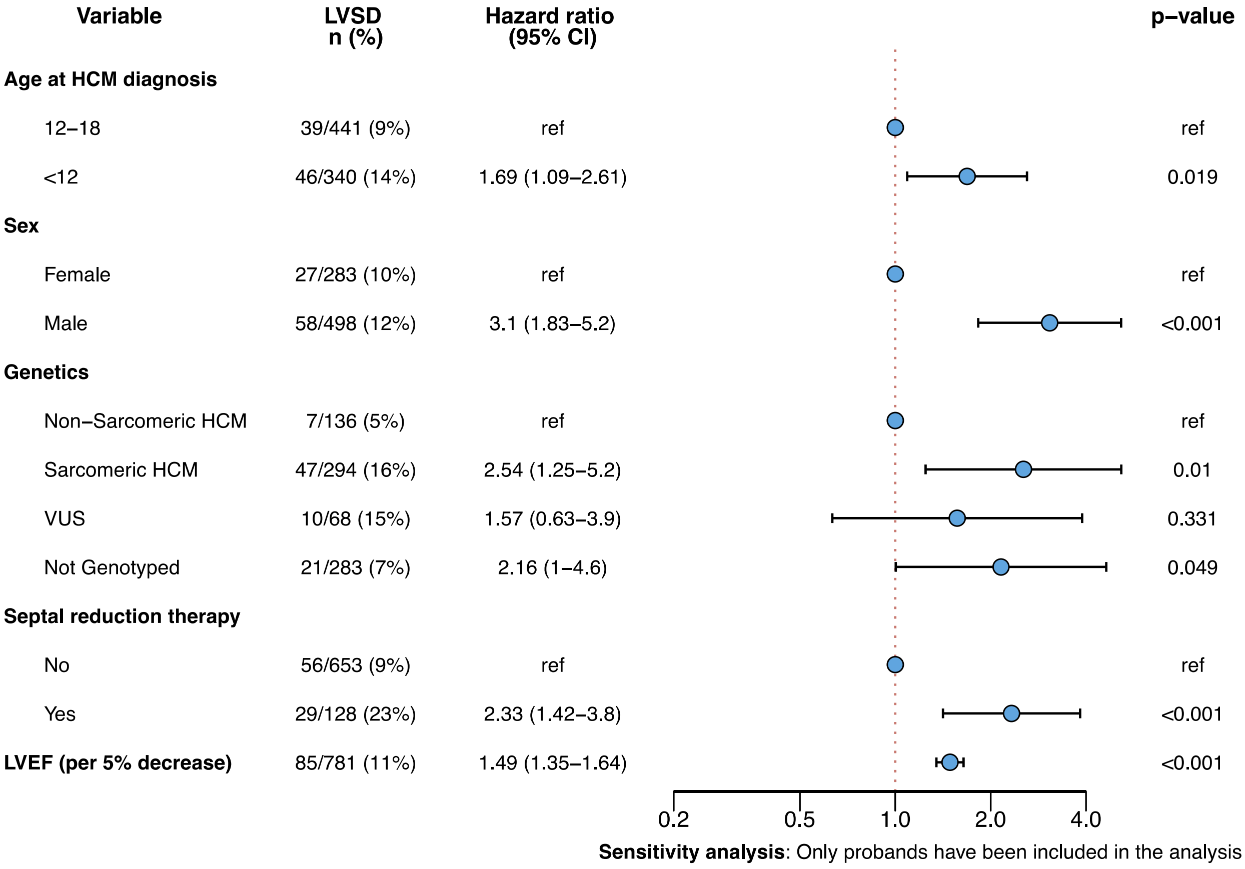

Supplementary Figure S8: Predictors of developing incident LVSD in patients with childhood-diagnosed HCM from the time of first SHaRe evaluation including probands only. Patients with LVSD at first evaluation or missing values on either LV wall thickness or ejection fraction at initial evaluation were excluded. VUS, variant of unknown significance.

Supplementary Figure S9: Cox model of the composite outcome – sensitivity analysis including only probands

Variable	Death, Txp, LVAD n (%)	Hazard ratio (95% CI)	p–value
**Time to LVSD from diagnosis**			
Per 5–year increase	57 / 137 (42 %)	0.82 (0.6 – 1.13)	0.23
**Sex**			
Male	32 / 93 (34 %)	ref	ref
Female	25 / 44 (57 %)	2.57 (1.38 – 4.78)	0.003
**Genetics**			
Non–Sarcomeric HCM	10 / 17 (59 %)	ref	ref
Sarcomeric HCM	30 / 70 (43 %)	0.52 (0.21 – 1.27)	0.154
VUS	2 / 10 (20 %)	0.33 (0.06 – 1.71)	0.187
Not Genotyped	15 / 40 (38 %)	0.7 (0.28 – 1.78)	0.455
**LVEF < 35%**			
No	42 / 113 (37 %)	ref	ref
Yes	15 / 24 (62 %)	3.75 (2.1 – 6.69)	<0.001
**Septal reduction therapy**			
No	45 / 96 (47 %)	ref	ref
Yes	12 / 41 (29 %)	0.71 (0.35 – 1.46)	0.355

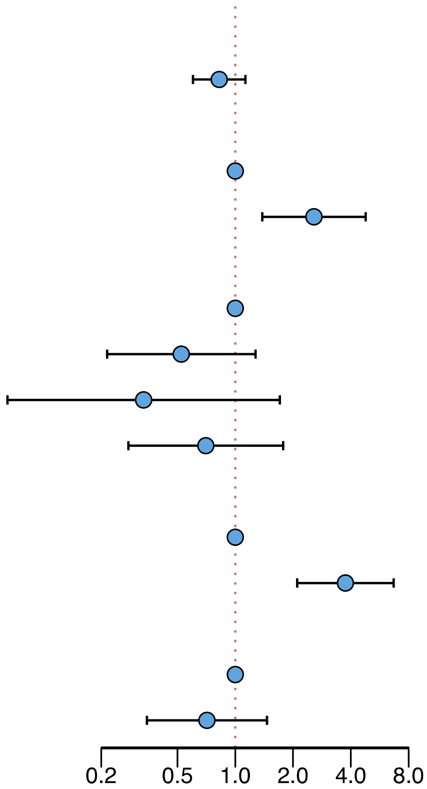

Sensitivity analysis: Only probands have been included in the analysis

Supplementary Figure S9: Predictors of the primary outcome of death, cardiac transplantation (Txp), or left ventricular device (LVAD) implantation in patients diagnosed with HCM during childhood who develop LVSD including probands only. VUS, variant of unknown significance.

Supplemental Tables:

Supplementary Table S1: Clinical outcomes of patients with childhood-diagnosed hypertrophic cardiomyopathy, stratified by left ventricular systolic dysfunction (LVSD) status. The "All LVSD" group represents patients with both prevalent and incident LVSD.

**Characteristic**	**All LVSD, N = 148**[1]	**No LVSD, N = 858**[1]
**Age at HCM diagnosis**	11.9 (7.6, 14.9)	12.9 (8.1, 15.4)
**Left ventricular assist device**	5 (3.4%)	0 (0%)
**Cardiac transplantation***	38 (26%)	19 (2.2%)
**All-cause mortality**	24 (16%)	30 (3.5%)
**Causes of death***		
Heart failure	8 (33%)	3 (10%)
Non-cardiovascular death	3 (12%)	4 (13%)
Not Recorded	2 (8.3%)	0 (0%)
Other cardiovascular death	2 (8.3%)	8 (27%)
Sudden cardiac death	9 (38%)	15 (50%)

[1]Median (IQR); n (%)

*This includes 2 patients who died after transplantation; 4 who underwent LVAD implantation prior to transplant, and 2 who were transplanted prior to initial SHaRe visit.

Supplementary Table S2: Characteristics of patients diagnosed with HCM during childhood or adulthood with and without prevalent LVSD.

Characteristic	No LVSD, Adult-Dx, N = 6,549	No LVSD, Childhood-Dx, N = 954	Prevalent LVSD, Adult-Dx, N = 192	Prevalent LVSD, Childhood-Dx, N = 56
**Female sex**	2,619 (40%)	339 (36%)	83 (43%)	19 (34%)
**Age at HCM diagnosis, years**	49.6 (37.4 to 60.6)	12.7 (8.1 to 15.3)	44.1 (31.9 to 57.0)	12.5 (7.1 to 15.2)
**Age at first SHaRe evaluation, years**	53 (42, 64)	15 (11, 21)	54 (46, 64)	31 (15, 41)
**Proband**	5,868 (91%)	831 (88%)	174 (91%)	52 (93%)
**NYHA functional class**				
1	2,658 (48%)	393 (68%)	47 (31%)	12 (29%)
2	2,008 (36%)	150 (26%)	55 (36%)	15 (36%)
3	829 (15%)	33 (5.7%)	44 (29%)	12 (29%)
4	41 (0.7%)	5 (0.9%)	8 (5.2%)	3 (7.1%)
Unknown	1,013	373	38	14
**Self-Reported Race**				
White	5,441 (83%)	772 (81%)	165 (86%)	47 (84%)
Black	278 (4.2%)	56 (5.9%)	7 (3.6%)	2 (3.6%)
Asian	211 (3.2%)	25 (2.6%)	9 (4.7%)	1 (1.8%)
Other or Not Reported	619 (9.5%)	101 (11%)	11 (5.7%)	6 (11%)
**Initial LVEF (%)**	65.8 ± 7.8	66.5 ± 8.6	38.2 ± 8.4	37.8 ± 9.1
**Obstruction Present**	2,312 (42%)	140 (25%)	12 (9.9%)	2 (9.5%)
Unknown	1,009	395	71	35
**LA diameter, mm**	40.9 ± 10.2	35.7 ± 10.8	47.1 ± 12.3	43.2 ± 13.5
**Maximal LV wall thickness, mm**	17.0 (14.0 to 21.0)	16.5 (12.0 to 23.0)	15.0 (12.0 to 18.0)	14.0 (12.0 to 20.4)
**Genetic Status**				
Sarcomeric HCM	1,954 (43%)	393 (63%)	73 (58%)	26 (72%)
Non-Sarcomeric HCM	2,115 (47%)	151 (24%)	38 (30%)	10 (28%)
VUS	437 (9.7%)	84 (13%)	15 (12%)	0 (0%)
Unknown	2,043	326	66	20
**Genes with P/LP variants**				
MYBPC3	1,189 (60%)	159 (40%)	41 (56%)	7 (27%)
MYH7	514 (26%)	170 (43%)	17 (23%)	12 (46%)
TNNI3	80 (4.1%)	15 (3.8%)	3 (4.1%)	2 (7.7%)
TNNT2	72 (3.6%)	23 (5.8%)	5 (6.8%)	0 (0%)
other	56 (2.8%)	11 (2.8%)	1 (1.4%)	2 (7.7%)
Multiple P/LP	43 (2.2%)	10 (2.5%)	3 (4.1%)	2 (7.7%)
TPM1	21 (1.1%)	6 (1.5%)	3 (4.1%)	1 (3.8%)
Unknown	4,574	560	119	30

n (%); Median (25% to 75%); Median (IQR); Mean ± SD

Dx, Diagnosis; LVEF, left ventricular ejection fraction; LA, left atrial; NYHA, New York Heart Association; P/LP, pathogenic/likely pathogenic; VUS, variant of uncertain significance
